# Molecular Recognition of Paired Receptors in the Immune System

**DOI:** 10.3389/fmicb.2012.00429

**Published:** 2012-12-31

**Authors:** Kimiko Kuroki, Atsushi Furukawa, Katsumi Maenaka

**Affiliations:** ^1^Laboratory of Biomolecular Science, Faculty of Pharmaceutical Sciences, Hokkaido UniversitySapporo, Japan; ^2^Core Research for Evolutional Sciences and Technology, Japan Science and Technology AgencySaitama, Japan

**Keywords:** paired receptor, immunoglobulin-like receptor, c-type lectin-like receptor, infectious diseases, tumorigenesis, ITIM, ITAM, structural biology

## Abstract

Cell surface receptors are responsible for regulating cellular function on the front line, the cell membrane. Interestingly, accumulating evidence clearly reveals that the members of cell surface receptor families have very similar extracellular ligand-binding regions but opposite signaling systems, either inhibitory or stimulatory. These receptors are designated as paired receptors. Paired receptors often recognize not only physiological ligands but also non-self ligands, such as viral and bacterial products, to fight infections. In this review, we introduce several representative examples of paired receptors, focusing on two major structural superfamilies, the immunoglobulin-like and the C-type lectin-like receptors, and explain how these receptors distinguish self and non-self ligands to maintain homeostasis in the immune system. We further discuss the evolutionary aspects of these receptors as well as the potential drug targets for regulating diseases.

## Paired Receptors

Paired receptors are related membrane proteins that are mainly expressed on immune cells. They share significantly conserved amino acid sequences within the extracellular domains, but have both activating and inhibitory members. The inhibitory receptors possess the immunoreceptor tyrosine-based inhibitory motif (ITIM) within the cytoplasmic region. In contrast, the activating receptors have short cytoplasmic regions and a positively charged residue (Arg or Lys) in the transmembrane domain to associate with an adaptor protein possessing the immunoreceptor tyrosine-based activation motif (ITAM). They are located in small gene clusters on a chromosome, and are usually expressed on overlapping immune cells.

Although most of the inhibitory receptors can bind to the endogenous ligands, it has been somewhat more difficult to identify the ligands of the activating receptors (Table 1), and their functions have not been determined. The typical immune cell receptor-ligand interaction is quite weak (*K*_d_ in the μM range; Table 2), with fast association and dissociation. When the activating and inhibitory receptors specifically recognize the same ligand, the inhibitory receptors usually have higher affinity (Table 2). This indicates that the inhibitory receptors function in the maintenance of immunological tolerance by the recognition of self-ligands, such as major histocompatibility complex class I molecules [MHCIs, also referred to as human leukocyte antigen class I molecules (HLAIs)]. Especially, in natural killer (NK) cells, which lack a gene arrangement system to recognize foreign antigens, the inhibitory receptors for MHCIs recognize and eliminate cells that fail to express MHCIs, due to viral infections or tumor formation. This hypothetical mechanism is known as the “missing-self hypothesis” (Ljunggren and Karre, 1990). Based on this hypothesis, Valiante et al. (1997) suggested a model in which each NK cell expresses at least one inhibitory receptor for the classical MHCIs, to avoid killing healthy self-cells. However, a subpopulation of human NK cells that lack inhibitory receptors for self-MHCIs was identified (Cooley et al., 2007). Furthermore, there are populations of “licensed” and “unlicensed” NK cells, which are exposed and not exposed to self ligands for inhibitory receptors, respectively. Unlicensed NK cells are basically hyporesponsive, but seem to have important roles in tumor elimination and viral clearance.

**Table 1:** **Paired receptors and their ligands**.

Receptor	Function	Endogenous ligand	Non-self ligand
KIR2DL1	Inhibitory	HLA-C (group 2)	
KIR2DL2	Inhibitory	HLA-C (group 1, a subset of group 2), some HLA-B	
KIR2DL3	Inhibitory	HLA-C (group 1, a subset of group 2), some HLA-B	
KIR2DL4	Activating?	HLA-G	
KIR2DL5	Inhibitory	?	
KIR3DL1	Inhibitory	HLA-Bw4, a subset of HLA-A	
KIR3DL2	Inhibitory	HLA-A3, A11	CpG ODN
KIR3DL3	Inhibitory	?	
KIR2DS1	Activating	HLA-C (group 2)	
KIR2DS2	Activating	HLA-C (group 1)?	
KIR2DS3	Activating	HLA-C (group 2)?	
KIR2DS4	Activating	A subset of HLA-Cw4, A11	
KIR2DS5	Activating	?	
KIR3DS1	Activating	HLA-Bw4, a subset of HLA-A?	
LILRB1	Inhibitory	HLA-A, B, C, E, F, G	CMV UL18
LILRB2	Inhibitory	HLA-A, B, C, E, F, G, ANGPTLs (Zheng et al., 2012)	
LILRB3-5	Inhibitory	?	
LILRA1	Activating	HLA-B27, HLA-CfHC	
LILRA2, 4–6	Activating	?	
LILRA3	Soluble	HLA-CfHC, HLA-A, HLA-G	
PIR-B	Inhibitory	MHCI (Nakamura et al., 2004)	
		Nogo, MAG, OMgp (Atwal et al., 2008)	
		ANGPTLs (Zheng et al., 2012)	
PIR-A	Activating	MHCI (Nakamura et al., 2004)	
PILRα	Inhibitory	CD99, PANP, NPDC1, COLEC12	HSV-1 gB
PILRβ	Activating	CD99	HSV-1 gB
SIRPα	Inhibitory	CD47, SP-A, SP-D	
SIRPβ	Activating	SP-D	
SIRPγ	No signal	CD47	
DCIR	Inhibitory	?	
DCAR	Activating	?	
NKRP1-A	Activating	LLT1	
NKRP1-D	Inhibitory	Clrb (Iizuka et al., 2003)	
NKRP1-C, F	Activating	Clrg (Iizuka et al., 2003)	
Ly49A, C, I, etc	Inhibitory	H-2	CMV m157
Ly49D, H, etc	Activating		CMV m157, others?
CD94/NKG2A	Inhibitory	HLA-E (Qa-1)	
CD94/NKG2C, E	Activating	HLA-E (Qa-1)	Others?
MAIR-I	Inhibitory	?	
MAIR-II	Activating	?	
CD200R1	Inhibitory	CD200 (Wright et al., 2003)	
CD200R3,4	Activating	?	

*ANGPTLs, angiopoietin-like proteins; fHC, free heavy chain; Nogo, neurite outgrowth inhibitor; MAG, myelin-associated glycoprotein; OMgp, oligodendrocyte myelin glycoprotein; Clrb, C-type lectin-related molecule b*.

**Table 2:** **Examples of binding affinities of receptor-ligand interactions**.

Receptor	Ligand	*K*_d_ (μM)	Reference
LILRB1	HLA-G1	2.0	Shiroishi et al. (2003)
LILRB1	HLA-B35	8.8	Shiroishi et al. (2003)
LILRB1	HLA-Cw4	6.5	Shiroishi et al. (2003)
LILRB1	UL18	0.0021	Chapman et al. (1999)
LILRB2	HLA-A11	45	Shiroishi et al. (2003)
LILRB2	HLA-G1	4.8	Shiroishi et al. (2003)
LILRB2	HLA-B35	26	Shiroishi et al. (2003)
LILRB2	HLA-Cw4	14	Shiroishi et al. (2003)
LILRB2	HLA-Cw7	26	Shiroishi et al. (2003)
KIR2DL1	HLA-Cw4	7.2	Stewart et al. (2005)
KIR2DS1	HLA-Cw4	30	Stewart et al. (2005)
KIR2DL3	HLA-Cw7	7.0	Maenaka et al. (1999)
KIR3DS1	HLA-B27	7.0	Li et al. (2010)
PILRα	CD99	2.2	Tabata et al. (2008)
PILRβ	CD99	85	Tabata et al. (2008)
SIRPα	CD47	∼2.0	Brooke et al. (2004)
SIRPγ	CD47	∼23	Brooke et al. (2004)
NKRP1	LLT1	48	Kamishikiryo et al. (2011)
NKG2A/CD94	HLA-E	0.8–12.4	Kaiser et al. (2008)
NKG2C/CD94	HLA-E	5.2–18.2	Kaiser et al. (2008)

The immune system is considered to be tightly regulated by the balance between the activating and inhibitory signals through these paired receptors, and dysregulation of this balance often causes autoimmunity, allergy, and various infectious diseases.

## Immunoglobulin-Like Receptors

The immunoglobulin (Ig)-like receptors include the killer cell Ig-like receptors (KIRs), leukocyte Ig-like receptors (LILRs), murine paired Ig-like receptors (PIRs), Fc receptor, leukocyte-associated inhibitory receptors (LAIRs), NKp46, and so on. They have several conserved extracellular domains possessing a characteristic Ig-fold, consisting of 70–110 amino acids with a sandwich-like structure formed by two sheets of antiparallel β strands. A large number of Ig-like receptor genes are located within the leukocyte receptor complex (LRC) on human chromosome 19 and on mouse chromosome 7, the syntenic region in the mouse. Here, we discuss the ligands and the molecular recognition of several Ig-like receptors.

## Killer Cell Immunoglobulin-Like Receptors

**Figure 1:**
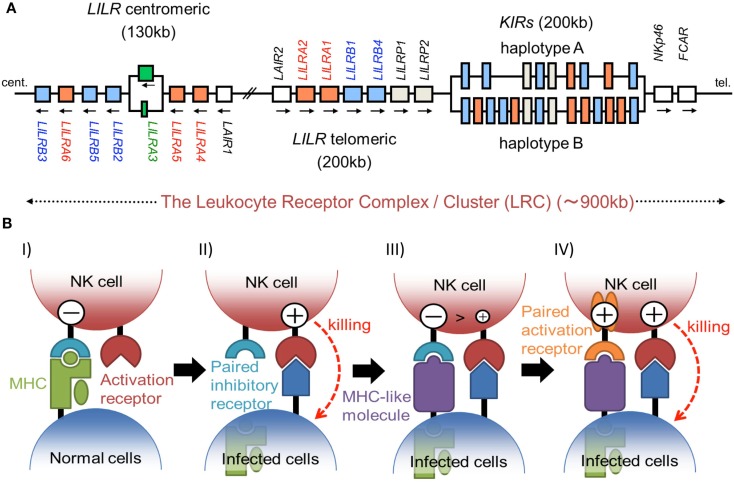
**(A)** Schematic representation of the LRC on human chromosome 19q13.4. A large number of Ig-like receptor genes, including two clusters of *LILR* loci and a cluster of *KIR* loci, are encoded within the LRC. Arrows indicate the direction of transcription for each gene. These loci have evolved by multiple duplications, and the two *LILR* clusters are likely to have been generated by the inverse duplication of an ancient one. **(B)** The hypothesis of paired receptor family evolution. I) The NK cell possesses at least one inhibitory receptor (cyan), and the inhibitory signals through it protect the normal self-cells from NK cell killing. II) In the infected cells, the low level expression of MHCIs induces the NK killing, in a system called the “missing-self hypothesis.” III) In order to escape the host NK cytotoxicity, some viruses acquired the expression of MHCI-like molecules (purple), which bind to the inhibitory receptors. IV) On the other hand, NK cells express activation receptors (orange), which evolved from the related inhibitory receptors to trigger NK cell activation.

**Figure 2:**
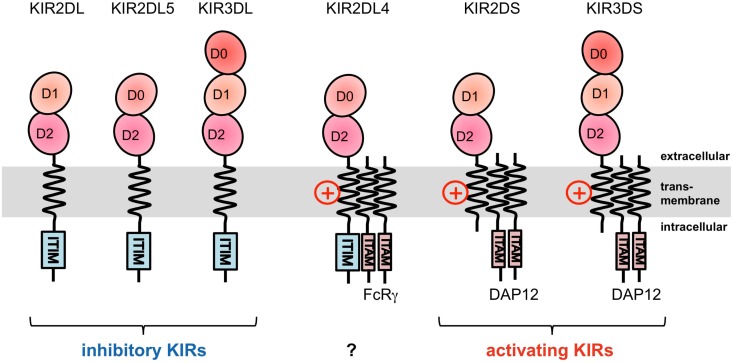
**Domain configuration of the KIRs**. The extracellular Ig-like domain is classified into three types, D0, D1, and D2, dependent on the sequence homology. KIR2DL4 possesses an ITIM motif, but also associates with the FcRγ chain.

The KIRs are expressed on NK cells and some subsets of T cells, and consist of 15 functional inhibitory (KIR2DL and KIR3DL) and activating (KIR2DS and KIR3DS) receptors. The *KIRs* are divided into two major haplotypes (haplotype A and haplotype B) and are encoded together with the *LILRs* (described later), forming a gene cluster within the LRC (Figure 1A). The *KIR* family is highly polymorphic, with not only nucleotide sequence polymorphisms but also the presence/absence of each locus. The KIRs basically recognize the classical MHCIs (HLA-A, -B, or -C) in an allele-specific fashion. The KIRs are classified into two structural groups, KIR2D and KIR3D, which have two and three Ig-like domains (D1–D2, D0–D2, or D0–D1–D2) in the extracellular region, respectively (Figure 2).

**Figure 3:**
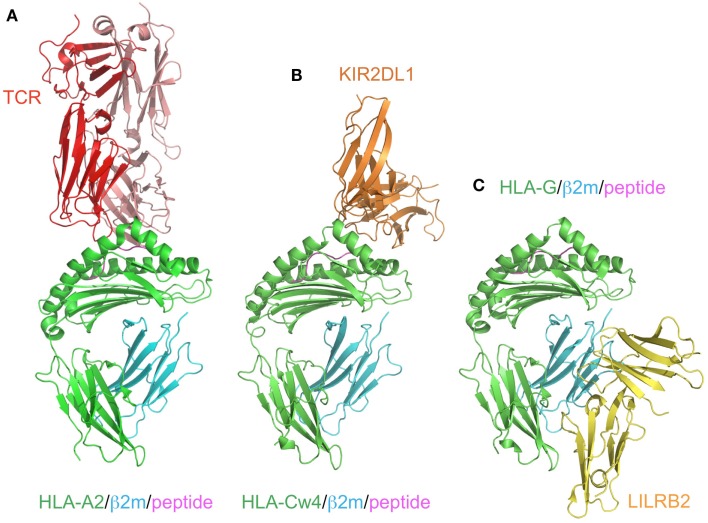
**Comparison of the recognition modes of the MHCI/MHCI receptors**. The complex structures of HLA-A2 and TCR (red; A, PDB ID:2VLR), HLA-Cw4 and KIR2DL1 (orange; B, PDB ID:1IM9), and HLA-G and LILRB2 (yellow; C, PDB ID: 2DYP). The MHCIs (heavy chain in green, β2m in cyan, peptide in magenta) are recognized in different manners. **(A)** TCR binds to the center of the peptide and the α1–α3 domain of HLA-A2. **(B)** KIR2DL1 binds to both the α1 and α2 helices of HLA and the C-terminal end of the peptide. This binding region contains the 77N/S and 80K/N residues, which determine the ligand specificity. **(C)** LILRB2 binds to the α3 domain and β2m, which are conserved regions among the MHCIs.

KIR2DL1 specifically binds to HLA–C group 2 molecules (Asn77 and Lys80), while KIR2DL2/2DL3 bind to HLA–C group 1 molecules (Ser77 and Asn80; Parham, 2005). The ligands of the KIR2DSs reportedly recognize the same MHCI molecules as those bound by their related inhibitory KIRs (Parham, 2005). In contrast to the T cell receptors (TCRs), which recognize a wide area of the bound peptide and its surrounding area (α1 and α2 helices) of the MHCIs (Figure 3A), KIR2Ds bind to the C-terminal site of the bound peptide (Maenaka et al., 1999; Boyington et al., 2000; Fan et al., 2001). This peptide-dependent recognition (Figure 3B) is relatively less specific than that of the TCRs.

KIR3DL1 binds to HLA–B with the Bw4 epitope, determined by amino acid positions 77–83 (Parham, 2005). KIR3DL2 recognizes some HLA-A alleles (Parham, 2005). A recent structural study of the KIR3D-MHCI complex revealed that, while the additional N-terminal Ig-like domain (D0) bound to the bottom of the α2 and α3 domains, the C-terminal two Ig-like domains (D1 and D2) exhibited essentially the same binding mode as the KIR2Ds (Vivian et al., 2011). This explains the common peptide-dependent MHCI recognition of the KIR members. On the other hand, the KIRs have both inhibitory and activating members, and basically the activating KIRs exhibit much lower or non-detectable affinity to MHCIs than the inhibitory ones. It is potentially possible that some peptides can bind more strongly to the activating KIRs than the inhibitory ones, even though Stewart et al. (2005) demonstrated that most (or maybe all) peptides did not follow this characterization. Interestingly, recent reports demonstrated that the peptide mutations are likely to play a pivotal role in regulating human immunodeficiency virus (HIV) infection, by mediating KIR recognition (Thananchai et al., 2009; Alter et al., 2011). This illustrates some similarity between the KIR and TCR functions, but in the opposite way (KIR may have a more inhibitory role, but that of TCR is stimulatory).

Unexpectedly, KIR3DL2 was recently found to bind to the microbial CpG oligonucleotide (ODN), and the D0 domain is primarily involved in this recognition. The internalization of the KIR3DL2-ODN complex causes the activation of NK cells through toll-like receptor 9 (TLR9) signals (Sivori et al., 2010). As a novel ligand recognition system, KIR would directly bind to microorganisms and take advantage of non-self ligands in order to regulate the host immune system.

Recently, KIR2DS2 and KIRDS4 were reported to be up-regulated after hematopoietic cell transportation, and their up-regulations were significant in cytomegalovirus viremia (Gallez-Hawkins et al., 2011). This suggested that the expression levels of KIRs are also important for controlling NK cell or T cell function. Furthermore, the KIR expression on cord blood T cells was induced during a human congenital infection with *Trypanosoma cruzi*, possibly by epigenetic mechanisms.

## Leukocyte Immunoglobulin-Like Receptors

The Leukocyte immunoglobulin-like receptor (LILR; LIR, ILT, CD85) family was initially identified as the cellular counter structure to the viral UL18 protein, an MHCI homolog expressed by human cytomegalovirus (hCMV; Cosman et al., 1997). To date, 13 *LILR* family genes, including two pseudogenes (*LILRP1* and *LILRP2*), have been identified. The LILR family members can be divided into three classes: the inhibitory LILRs (LILRB1, -B2, -B3, -B4, -B5) with ITIM-like sequences, the activating LILRs (LILRA1, -A2, -A4, -A5, -A6) with a positively charged Arg residue in the transmembrane domain pairing with the FcRγ chain containing an ITAM, and the soluble LILR (LILRA3) with no transmembrane region. The LILRs have a broad cellular distribution that includes NK, T, and B lymphocytes, as well as myelomonocytic cells such as macrophages, mast cells, and dendritic cells.

The *LILR* family genes are encoded within the LRC on human chromosome 19q13 (Figure 1A). In the syntenic region of the mouse, at the proximal end of chromosome 7, the *LILR* gene-orthologous *Pir-a* and *Pir-b* are located. There are two clusters of *LILR* genes (*LILR* centromeric and *LILR* telomeric) that are transcribed in opposite directions (Figure 1A). The similarity of their amino acid sequences with that encoded by *KIR* suggests that the *LILRs* and *KIRs* are related by a recent gene duplication event. *LILR* genes have been found in a wide variety of species and are more stable in number, in contrast to the *KIR* genes. However, in addition to the deletion of the *LILRA3* gene which is absent in some individuals, recent studies revealed that *LILRA3* and *LILRA6* show high levels of genetic diversity, with decreased copy numbers in Asians (Hirayasu et al., 2008) and increased variability in Africans (Sudmant et al., 2010).

Although the KIR and LILR protein families are structurally and functionally comparable, there are some distinguishing characteristics. LILRB1 and LILRB2 bind to a variety of MHCIs through two N-terminal extracellular domains (D1 and D2; Borges et al., 1997; Colonna et al., 1997; Cosman et al., 1997). They recognize MHCIs on target cells to mediate inhibitory signals, to prevent the killing of normal cells expressing MHCIs. In other words, abnormal cells expressing few or no MHCIs can activate the cellular function of LILRB1/B2-positive leukocytes, due to the lack of the LILRB1/B2-mediated inhibitory signal. LILRA1 and LILRA3 also bind to some MHCIs (Allen et al., 2001; Ryu et al., 2011). Whereas most KIRs recognize discrete polymorphic epitopes within the α1 and α2 domains of MHCIs (Figure 3B), a characteristic consistent with their narrow binding specificities, LILRB1/B2 binding is mediated via a site in the conserved α3 and β2m domains of the MHCI molecule (Figure 3C; Willcox et al., 2003; Shiroishi et al., 2006). Moreover, LILRB1/B2 effectively compete with CD8 for MHCI binding, and modulate CD8^+^ T cell activation by blocking CD8 binding as well as by recruiting inhibitory molecules through their ITIMs (Shiroishi et al., 2003). This system was also observed in the binding of the mouse LILRB homolog, PIR-B, to MHCIs (Shiroishi et al., 2003).

**Figure 4:**
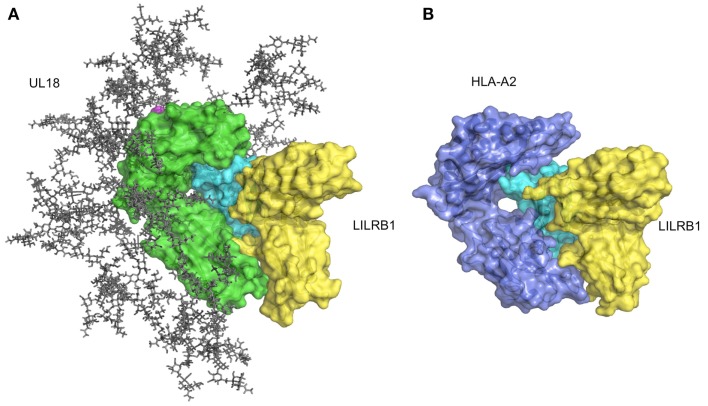
**Structure of UL18/LILRB1 and comparison with the HLA-A2/LILRB1 complex**. **(A)** The crystal structure of the UL18/LILRB1 complex (UL18 in green, β2m in cyan, peptide in magenta, LILRB1 in yellow; PDB ID: 3D2U) with complex carbohydrate models attached to the 13 potential *N*-glycosylation sites. The α1–α2 domains recognized by TCR or KIRs are highly glycosylated, and steric hindrance inhibits effective interactions. Meanwhile, the interface with LILRB1 still is exposed. **(B)** The crystal structure of the HLA-A2/LILRB1 complex (HLA-A2 and peptide in purple, β2m in cyan, LILRB1 in yellow; PDB ID: 1P7Q).

As described above, LILRB1 also binds to the UL18 protein with much higher affinity than the MHCIs (Cosman et al., 1997), but LILRB2 and LILRA1 do not (Borges et al., 1997). UL18 is a highly glycosylated protein (Figure 4A) sharing 25% sequence identity with MHCIs (Beck and Barrell, 1988). Although the LILRB1/UL18 complex structure revealed that the binding mode was conserved with those of the LILRB1/MHCIs (Figures 4A,B), the residues within the α3 domain differed and created a more favorable binding region at the interface with LILRB1 (Yang and Bjorkman, 2008). Moreover, the 13 potential *N*-glycosylation sites effectively covered the interaction sites for the potential UL18 binding partners, including the α1–α2 domain binding to KIR and TCR and the part of α3 domain binding to CD8. On the other hand, only the binding region of LILIRB1 remains exposed (Figure 4A). These structural features demonstrated how the viral protein UL18 can effectively compete with the host ligands for LILRB1, to regulate the host’s immune response.

## Paired Type 2 Immunoglobulin-Like Receptors

The Paired type 2 immunoglobulin-like receptors (PILRs) are expressed mainly on immune cells and have one Ig-like domain in the extracellular region, with either an ITIM in the intracellular domain (inhibitory receptor, PILRα) or a positively charged amino acid in the transmembrane region associated with the activating subunit, DNAX activating protein of 12 kDa (DAP12; activating receptor, PILRβ; Fournier et al., 2000). The PILRs recognize sialylated *O*-linked sugar-modified mucin and mucin-like molecules, such as CD99 (Wang et al., 2008), PILR-associating neural protein (PANP; Kogure et al., 2011), and two newly identified ligands, neuronal differentiation and proliferation factor-1 (NPDC1), and collectin-12 (COLEC12; Sun et al., 2012), as physiological ligands. Notably, the PILR-ligand family is further expanding, as the report by Sun et al. (2012) described that the PILRs can also bind to immune cells that do not express any identified PILR-ligands. As mentioned above, PILRα shows higher affinity to these ligands than PILRβ, which is typical for paired receptors in the immune system (Table 2). On the other hand, recent reports revealed that PILRα is the receptor for herpes simplex virus 1 (HSV-1), by binding to its glycoprotein B (gB; Satoh et al., 2008). HSV-1 gB can utilize the inhibitory member, PILRα, as an entry receptor, and thus it is probably beneficial for HSV-1 infection. The PILRs recognize physiological and viral proteins in similar but non-identical manners, and notably exhibit both sugar- and peptide-dependent binding modes, which are quite unique for sugar-protein interactions. Even though the structural information about the PILRs is still lacking, mutagenesis studies suggested that the PILR-ligand recognition modes are somewhat similar to those of the sialic acid-binding Ig-like lectins (Siglec) family, which can bind to sialic acid (Tabata et al., 2008; Wang et al., 2008). However, the significant and unusual characteristic, that the PILRs cannot bind to either sugars or peptides only, and require both for binding, is largely unknown and rather unique. Future studies, especially crystallographic and NMR analyses, will clarify the molecular mechanisms of these binding systems.

## Signal Regulatory Proteins

The Signal regulatory proteins (SIRP) family has three members, SIRPα, SIRPβ, and SIRPγ. SIRPα (SHPS-1, BIT, CD172A) is broadly expressed on myeloid cells, such as neutrophils, macrophages, and dendritic cells, as well as on neurons (Adams et al., 1998). SIRPα interacts with CD47, which expresses in hemopoietic cells, epithelial cells, and endothelial cells, as well as in brain and mesenchymal cells, resulting in the transmission of the inhibitory signal through the SH2-domain-containing protein tyrosine phosphatases 1 and 2 (SHP1 and 2, respectively), and finally causes reduced phagocytosis activity in macrophages and cytokine production in various cells (Barclay and Brown, 2006). In this sense, SIRPα plays an important role in immune suppression. In contrast, SIRPβ can potentially generate activation signaling by associating with DAP12, but it cannot interact with CD47. In addition, SIRPγ binds to CD47 with 10-fold lower affinity as compared to SIRPα, but lacks a signaling motif (Barclay and Brown, 2006). An X-ray crystallographic analysis indicated that the difference in the binding affinities of the SIRPs with CD47 is due to the subtle differences in the loops, with direct and indirect effects (Hatherley et al., 2008).

The other function of the SIRPs is to bind with Surfactant Protein D (Sp-D). Sp-D is an important component of the pulmonary surfactant involved in host innate immunity, and is capable of binding most Gram-negative bacteria as well as several Gram-positive bacteria, leading to increased opsonization of bacteria. On the other hand, the binding of Sp-D to SIRPα transmits the immune suppression signals, resulting in decreased cytokine production (Gardai et al., 2003). Interestingly, the SIRPα binding to SP-D was competed with lipopolysaccharide (LPS; Fournier et al., 2012). These observations indicated that the anti-inflammation signals through SIRPα are present in the absence of pathogens, and once pathogens are present, Sp-D binds preferably to LPS or other bacterial carbohydrates, and then induces the host innate immunity. Under such conditions, the absence of ligand binding to SIRPα also elicits an increase in inflammation (Gardai et al., 2003). One recent report demonstrated that SIRPβ also binds to Sp-D, but with slightly lower affinity as compared to SIRPα (Fournier et al., 2012). These results suggested that SIRPs exhibit self/non-self discrimination and cooperatively modulate the immuneresponses.

## C-Type Lectin-Like Receptors

C-type lectin-like receptors (CLRs) are expressed on the cell surface of various immune cells, to regulate the innate immune systems. The term “C-type lectin” means Ca^2+^ dependent carbohydrate-binding lectin. The CLRs contain a conserved motif, either EPN (Glu-Pro-Asn) or QPD (Gln-Pro-Asp). This motif is located in a structurally conserved loop, which is stabilized by a disulfide bond with another conserved loop. The EPN motif confers specificity for mannose-based ligands, whereas the QPD motif is typical of the galactose-specific Carbohydrate Recognition Domain (CRD; Zelensky and Gready, 2005). The carbonyl side chains of these amino acid residues coordinate Ca^2+^, form hydrogen bonds with individual monosaccharides, and determine binding specificity. Due to the versatile recognition ability of the CLRs (described in below), they are known as pathogen associated molecular patterns (PAMPs) recognition receptors. The CLRs are primarily involved in detecting pathogens and subsequently triggering signaling pathways to evoke various immune reactions. Meanwhile, a few CLRs are known to act as immuno-repressive receptors. Interestingly, it was demonstrated that Macrophage inducible C-type lectin (Mincle, also called CLEC4E) recognized not only the sugar components from pathogens through the CRD but also proteins from pathogens or self, through sites other than the CRD (discussed in detail below). Here, we describe the detailed functions and structures of several CLRs, including orphan CLRs that have yet to be characterized, to shed light on the molecular mechanisms of the ligand recognition by the paired-receptor-type CLRs.

## CD94/NKG2

The CD94/NKG2 receptors are expressed on the surfaces of a greater part of NK cells and some subsets of CD8^+^ T cells, and belong to the CLRs. While CD94 is encoded by a single gene and has extremely low polymorphism, the NKG2 molecules have five isotypes (NKG2A, C, D, E, and F) and two splice variants, NKG2B and NKG2H, derived NKG2A and NKG2E, respectively. Five NKG2 molecules (NKG2A, B, C, E, and H) have been shown to form disulfide-linked heterodimers with CD94. CD94/NKG2A and B mediate inhibitory signaling through the ITIM of the cytosolic region in NKG2s. In contrast, CD94/NKG2C, E, and H induce activation signaling through the interaction between a Lys residue within their transmembrane region and a negatively charged residue in the ITAM-containing adaptor molecule, DAP12.

The ligand of most CD94/NKG2s is the non-classical MHCI, HLA-E (Borrego et al., 1998; Braud et al., 1998; Lee et al., 1998; Brooks et al., 1999; Table 1). HLA-E is expressed in all nucleated cells and has few polymorphisms, as compared to the classical MHCI. HLA-E mainly presents the peptides derived from the leader sequences of other MHCIs. Therefore, NK cells monitor the MHCI expression level through the interactions between the inhibitory NKG2A/CD94s and HLA-E. Thus, NK cells interact with healthy cells, which show normal expression levels of HLA-E, resulting in the inhibition of NK cell killing activity.

**Figure 5:**
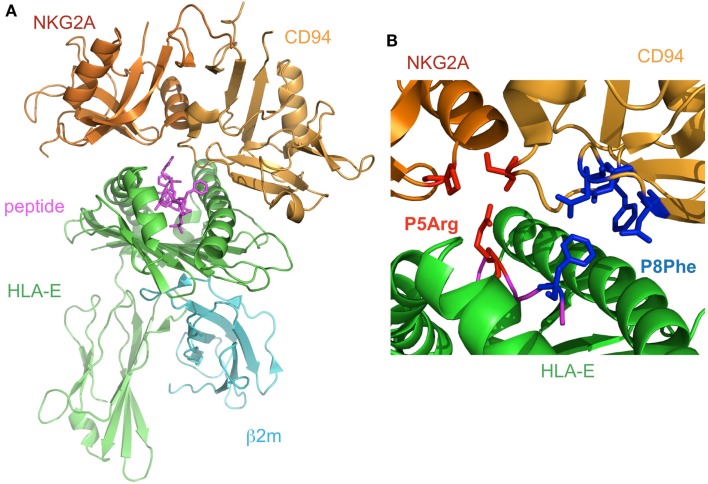
**The complex structure of NKG2A/CD94 and HLA-E**. **(A)** The overall structure of the NKG2A/CD94/HLA-E complex (PDB ID: 3CDG). NKG2A/CD94 recognizes the α1–α2 domain of HLA-E containing a peptide. **(B)** The interface of NKG2A/CD94 and HLA-E. The residues interacting with P5Arg of the peptide are depicted by red stick models, and P8Phe is shown by a blue stick model.

The structure of CD94/NKG2A in complex with HLA-E loaded with an HLA-G derived peptide has been determined (Kaiser et al., 2008; Petrie et al., 2008; Figure 5A). NKG2A and CD94 interact with the α1 and α2 helices of the peptide-binding region of HLA-E, respectively, with charge complementarity. The presented peptide is also recognized by CD94/NKG2A, while CD94 is mainly recognized HLA-E and the peptide, as compared to NKG2A. The Arg (P5) and Phe (P8) residues of the peptides contribute to binding with CD94/NKG2A (Figure 5B). P5Arg is conserved in the leader sequence of MHCI, and its replacement with a Lys abolished the interaction with CD94/NKG2A. Hydrophobic amino acid residues, such as Phe and Leu, are conserved in P8 of the leader sequence, and their replacement with Lys led to a dramatic reduction in the binding ability of HLA-E with CD94/NKG2. These data indicated that the Arg in P5 and the hydrophobic amino acid residue in P8 are both indispensable for the interaction with CD94/NKG2A.

The interaction between CD94/NKG2 and HLA-E is associated with a wide range of diseases, such as virus infection and cancer. For instance, HCMV utilizes the host’s CD94/NKG2 system to grow. The leader sequence of the HCMV protein, UL40, is identical to that of HLA-Cw3. The CD94/NKG2A on NK cells recognizes the HLA-E associated peptide, which is derived from the leader sequence of the HCMV protein, on the infected cells, and therefore its interaction inhibits NK cells from attacking infected cells (Tomasec et al., 2000; Ulbrecht et al., 2000). In addition, in renal cell carcinoma-infiltrating NK cells, the expression level of CD94/NKG2A is relatively high. This led to the proposal that tumor cells control the expression level of CD94/NKG2A, and consequently the function of NK cells (Schleypen et al., 2003). It might be plausible that other intracellular pathogens can also utilize the CD94/NKG2A systems to escape from the host’s immune system.

## Dendritic Cell Immunoreceptor/Dendritic Cell Immunoactivating Receptor

Dendritic cell immunoreceptor (DCIR; CLEC4A) is one of the repressive CLRs. DCIR is expressed on the surface of various immune cells, such as dendritic cells, monocytes, macrophages, B cells and neutrophils (Bates et al., 1999). Although DCIR reportedly binds HIV-1, no physiological ligands for DCIR have been identified (Lambert et al., 2008). The ITIMs in the cytoplasmic tails of DCIR serve to recruit SHP1 or SHP2 after ligand binding (Richard et al., 2006). The Syk and Src kinases (i.e., Src, Fyn, and Hck), as well as the PKC-γ MAP kinases (i.e., Erk1/2 and p38), are reportedly involved in the subsequent signaling pathway and finally inhibit TLR8-mediated IL-12 and TNF-α production significantly (Lambert et al., 2011). However, the precise mechanism of this inhibition is still unknown.

DCIR expression on neutrophils was reportedly down-regulated by TNF-α, IL-1α, and LPS stimulation, but was not affected by anti-inflammatory stimuli, including IL-4, IL-10, and IL-13 (Bates et al., 1999; Richard et al., 2002). These results suggested that DCIR may be down-regulated during pathogen exposure and inflammation.

A recent study showed that *Dcir*^−/−^ mice developed joint abnormalities, such as swelling and redness, at an early age, and these abnormalities eventually progressed to joint deformity and ankylosis (Fujikado et al., 2008). The *Dcir*^−/−^ mice developed sialadenitis, which is characterized by the accumulation of lymphocytes in the interstitium and the destruction of the small duct associated with mononuclear cell infiltration. The number of activated CD4^+^ T cells, the expression of IL-4 and IL-10, and the production of IgG1 and IgG3 were increased in *Dcir*^−/−^ mice. Furthermore, in *Dcir*^−/−^ mice, stimulation with granulocyte-macrophage colony–stimulating factor (GM-CSF) activated the phosphorylation of STAT5 and effectively differentiated bone marrow-derived cells to dendritic cells, as compared to wild type mice. These results suggested that DCIR regulates the proliferation of dendritic cells and is involved in maintaining immune self-tolerance.

On the other hand, mouse DCIR shares substantial sequence homology (91% aminoacid identity) in the extracellular region with its activating counter member, Dendritic cell immunoactivating receptor (DCAR). DCAR is expressed similarly in tissues to DCIR, but its short cytoplasmic portion lacks a signaling motif such as an ITIM (Fujikado et al., 2008). Instead, an Arg residue is present in the transmembrane region of DCAR, which participates in the association with the FcRγ chain and finally activates immune cells. Neither the human ortholog nor the ligands for DCAR have been identified yet.

## NKR-P1 (CD161)

**Figure 6:**
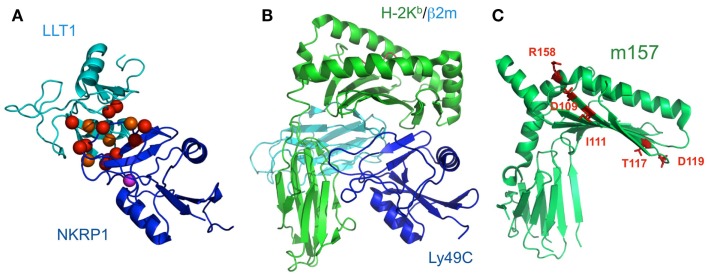
**Schematic model of LLT1 recognition by NKRP1 and comparison of the structures of the Ly49-MHCI complex and m157**. **(A)** The model structures of LLT1 (cyan) and NKRP1 (blue) are shown by ribbon models. Residues that may contribute to the interaction with LLT1 are shown as spheres, with detrimental effects in red, and modest effects in orange. Magenta spheres indicate the pair of residues that showed detrimental effects when mutated independently, but restored the binding when mutated simultaneously (Kamishikiryo et al., 2011). **(B)** The crystal structure of Ly49C with H-2K^b^ (H-2K^b^ in green, β2m in cyan, Ly49C in blue; PDB ID: 1P4L). Ly49C interacts with the β2m subunit and the α3 domain of H-2Kb, but not with the peptide-binding region. The crystal structure of m157 (PDB ID; 2NYK). **(C)** The mutation sites that identified the Ly49H binding residues are mapped and are shown in stick style (red). The residues did not overlap with the interface of the Ly49-MHCI complex structure.

NKR-P1 (CD161) is expressed on the surfaces of NK cells and subsets of T cells. Human NKR-P1 reportedly interacts with Lectin-like transcript-1 (LLT1, also called CLEC2D), which is expressed on many cell lines and on activated primary B cells (Aldemir et al., 2005; Rosen et al., 2005). The *NKR-P1* and *LLT1* genes are adjacent on human chromosome 12, and coordinately regulate the immune response. Rodents possess several *Nkrp1* genes for the activating (NKRP1-A, C, and F) and inhibitory (NKRP1B and G) receptors, while in contrast, there is only a single inhibitory *NKR-P1A* gene in human. LLT1 (also known as Clr-b in mouse) on target cells can inhibit NK cytotoxicity, by interacting with NKR-P1 on NK cells (Rosen et al., 2008). In rodents, NKR-P1C reportedly associates with the FcRγ chain, inducing not only cytotoxicity but also IFN-γ production. These data suggested that, although anon-self ligand has not been identified, the components of pathogens or dead cells may potentially stimulate NK cells through NKR-P1, and are eliminated by NK cells themselves and by other immune cells (Arase et al., 1997). The detailed molecular mechanisms of both the activating and inhibitory signaling pathways via NKR-P1 have not been characterized, but interestingly, the acid sphingomyelinase reportedly binds to the cytosolic region of NKR-P1 and participates in NK cell resistance to apoptosis (Pozo et al., 2006). We recently analyzed the molecular basis of the interaction between NKR-P1A and LLT1 (Figure 6A; Kamishikiryo et al., 2011), and proposed a model of the NKR-P1/LLT complex. The constructed model suggested that the membrane-distal head region of NKR-P1 is a new target to inhibit the NKR-P1-LLT1 interaction, potentially leading to the regulation of autoimmune and chronic inflammatory disorders.

## Other C-Type Lectin-Like Receptors

Other CLRs have been identified and functionally investigated. Macrophage inducible C-type lectin (Mincle, also called CLEC4E) is a type II transmembrane C-type lectin receptor expressed in macrophages, dendritic cells and monocytes. Mincle was initially identified as a gene up-regulated by LPS stimulation (Matsumoto et al., 1999). The first identified ligand of Mincle was the spliceosome associated protein 130 (SAP130), the endogenous protein released from necrotic cells (Yamasaki et al., 2008). Intriguingly, Mincle also reportedly recognized malassezia species and *Mycobacterium tuberculosis* (Ishikawa et al., 2009; Yamasaki et al., 2009). While the ligand structures of the malassezia species are not known, that of *M. tuberculosis* was identified as trehalose-1,1-dimycolate (TDM). The malassezia species and *M. tuberculosis* are both recognized through the CRD of Mincle. Following the binding of either SAP130, malassezia species or TDM, Mincle associates with the ITAM-bearing FcRγ chain. This association leads to the phosphorylation of the ITAM of the FcRγ chain and the subsequent recruitment of Syk, activating the caspase recruitment domain family, member 9 (CARD9)-mediated NF-κB signaling pathway to promote the expression of TNF and IL-6. A recent report revealed that Mincle would likely playa non-redundant role for T cell immune responses to infection by microbes (Schoenen et al., 2010).

The Dendritic Cell-Specific Intercellular adhesion molecule-3-Grabbing Non-integrin (DC-SIGN, also called CLEC4L and CD209) proteins have been extensively studied, due to their broad range recognition of pathogens and ligands from viruses to parasites, such as HIV-1, measles, dengue, SARS, *Helicobacter pylori*, *E. coli*, *Leishmania* spp., and *Schistosoma* egg (Sancho and Reis e Sousa, 2012). The binding of DC-SIGN with ligands from pathogens activates human myeloid dendritic cells through various pathways. Mannose-expressing *M. tuberculosis* and HIV-1 promote the activation of LARG and RhoA, which function as upstream activators of Raf-1 via DC-SIGN (Gringhuis et al., 2007, 2009; Hodges et al., 2007). This activation is mediated by the phosphorylation and acetylation of NF-κB subunit p65, which greatly enhances the transcriptional activity of NF-κB and results in the modulation of TLR4 signaling and the enhanced expression of IL-10, IL-12, and IL-6 (Gringhuis et al., 2007). In contrast, fucose-expressing pathogens, such as *H. pylori*, actively dissociated the KSR1–CNK–Raf-1 complex and enhanced the expression of IL-10, but down-regulated the expression of IL-12 and IL-6 in a Raf-1-independent, but LSP1-dependent, manner (Gringhuis et al., 2009). Notably, DC-SIGN cannot activate NF-κB by itself, and it modulates the p65 activity only when p65 is induced by another receptor upon the stimulation of mannose-expressing pathogens (Gringhuis et al., 2007). In summary, the signaling via DC-SIGN is tightly regulated by the characteristics of the ligands.

DC NK lectin group receptor-1 (DNGR, also called CLEC9A) was previously shown to function as a Syk-coupled C-type lectin receptor, to mediate the sensing of necrosis (Sancho et al., 2009). A recent report demonstrated that DNGR recognized exposed actin filaments from necrotic or damaged cells (Ahrens et al., 2012; Zhang et al., 2012). Mutational and crystallographic studies indicated that two exposed tryptophan residues in DNGR, which are conserved between human and mouse, are involved in the recognition of the actin filament. These residues are far from the C-type lectin domains, which function in stabilizing the structure of the protein, rather than being directly involved in the receptor-ligand interaction. So far, most of the ligands from pathogens are carbohydrate or carbohydrate-related products. However, several studies have clearly proved that CLR can interact with ligands through other regions than the C-type lectin domains. These results imply that non-carbohydrate ligands of the CLRs from pathogens will be discovered in the future.

## Discussion

Paired receptors are potentially dangerous, because activating receptors can disrupt homeostasis, thus threatening life. In the immune system, immune cells express such activating receptors on their cell surfaces; however, the education or licensing of these cells has been considered to require the expression of at least one inhibitory receptor to suppress inappropriate activation, at least in fully responsive mature cells. On the other hand, Arase et al. (2002) clearly showed excellent evidence for one paired receptor family, the Ly49 family. The susceptibility to mouse CMV (MCMV) depends on the mouse strain, and the protection of this virus is mediated by NK cells. Mice harboring only the inhibitory Ly49 family members, which bind to MHCIs (Held et al., 1996; Yu et al., 1996; Hanke et al., 1999) as well as the CMV MHCI homolog, m157 (Arase et al., 2002; Smith et al., 2002), cannot survive the CMV infection. However, other mice with the activating member, Ly49H, which binds to its m157 to activate the immune response, can evade CMV infection. Thus, m157 is the only known viral ligand binding to both inhibitory and activating receptors. Based on these observations, we have developed a scenario in which the activation receptor evolved from the related inhibitory receptor in response to selective pressure imposed by the pathogen, thus providing the presence of diversified, paired receptors (Figure 1B). In accordance with this scenario, the activating receptor, KIR2DS2, has a relic of the ITIM sequence, which is inactivated by the direct introduction of a stop codon (Arase and Lanier, 2004). This hypothesis suggests that other activating receptors will also recognize pathogen-derived ligands. Although m157 forms a typical MHCI-fold, it neither presents a peptide nor associates with β2m (Adams et al., 2007; Figures 6B,C). A comparison of the crystal structures of the Ly49/H-2 complexes and m157 revealed that a different interaction interface from that of Ly49/H-2 would exist upon the binding of Ly49s to m157.

Furthermore, Barclay and Hatherley (2008) proposed an elegant counterbalance theory. Accumulating mutations on paired receptors are often unrelated to the binding regions to physiological ligands, which may support the idea that these mutations are targeted to the non-self molecules of infectious microorganisms. As described above, the ancestral paired receptors are likely inhibitory to suppress undesired immune responses, but this is essentially beneficial for microorganisms, if they can be used as not only entry receptors but also inhibitory ones to facilitate immune evasion. The KIRs are considered to have co-evolved with their ligands, the MHCIs. For example, different human populations have a reciprocal relationship between the KIR and HLA-C frequencies (Hiby et al., 2004), and the frequency of the KIR2DL3-HLA-C1 combination could be reduced in populations highly exposed to malaria, by natural selection (Hirayasu et al., 2012). These observations suggested that the paired receptors and their ligands co-evolved. Therefore, the precise understanding of the on-going activating and inhibiting balance of paired receptors can provide insight into the extent of the importance of each set of paired receptors for immune defense. In this sense, the development of small molecular-weight compounds or biopharmaceuticals targeting paired receptors can more easily and finely regulate the immune responses, as advanced therapy for either infectious diseases or tumorigenesis. Especially, we believe that future accumulating information relating the genetic, molecular, and structural bases for paired receptors will greatly contribute to the development of novel therapies with fewer side effects.

## Conflict of Interest Statement

The authors declare that the research was conducted in the absence of any commercial or financial relationships that could be construed as a potential conflict of interest.
